# Curcumin and Diabetes: A Systematic Review

**DOI:** 10.1155/2013/636053

**Published:** 2013-11-24

**Authors:** Dong-wei Zhang, Min Fu, Si-Hua Gao, Jun-Li Liu

**Affiliations:** ^1^Diabetes Research Center, Beijing University of Chinese Medicine, Beijing 100029, China; ^2^Fraser Lab for Diabetes Research, McGill University Health Center, Montreal, Canada H3A 1A1

## Abstract

Turmeric (*Curcuma longa*), a rhizomatous herbaceous perennial plant of the ginger family, has been used for the treatment of diabetes in Ayurvedic and traditional Chinese medicine. The active component of turmeric, curcumin, has caught attention as a potential treatment for diabetes and its complications primarily because it is a relatively safe and inexpensive drug that reduces glycemia and hyperlipidemia in rodent models of diabetes. Here, we review the recent literature on the applications of curcumin for glycemia and diabetes-related liver disorders, adipocyte dysfunction, neuropathy, nephropathy, vascular diseases, pancreatic disorders, and other complications, and we also discuss its antioxidant and anti-inflammatory properties. The applications of additional curcuminoid compounds for diabetes prevention and treatment are also included in this paper. Finally, we mention the approaches that are currently being sought to generate a “super curcumin” through improvement of the bioavailability to bring this promising natural product to the forefront of diabetes therapeutics.

## 1. Introduction

**Figure 1:**
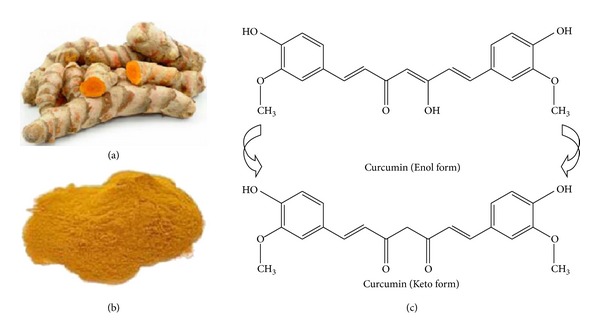
Turmeric, curcumin and its chemical structure. (a) The root of turmeric. (b) Crystallized powder of curcumin. Curcumin is thought to be the main active ingredient derived from the root of turmeric. (c) The enol and keto forms of curcumin are common structures of the drug. The enol form is more energetically stable in the solid phase and in solution [6]. Figures 1(a) and 1(b) are from http://www.skinvitality.ca/blog/2012/06/curcumin-cancer-treatment#.UnGdC7KBSnQ and http://en.wikipedia.org/wiki/Curcumin.

Natural products have received considerable attention for the management of diabetes and its complications [1–3] which have reached epidemic levels worldwide [4]. The spice turmeric, which is derived from the root of the plant *Curcuma longa*, has been described as a treatment for diabetes in Ayurvedic [5] and traditional Chinese medicine for thousands of years (Figure 1).

The most active component of turmeric, curcumin, has caught scientific attention as a potential therapeutic agent in experimental diabetes and for the treatment of the complications of diabetes patients [7], primarily because it is effective in reducing glycemia and hyperlipidemia in rodent models and is relatively inexpensive and safe [8–10]. The structure of curcumin (Figure 1(c)), shown to be a diferuloylmethane, was resolved by Lampe and Milobedeska in 1910 [11]. We retrieved more than 200 publications with the search term “*curcumin and diabetes*” from the MEDLINE database in 2013. The first paper that described an effect of curcumin related to diabetes described a blood glucose lowering effect of the drug in one diabetic individual only and was published in 1972 [12]. Curcumin has been since extensively studied in experimental animal models of diabetes and in a few clinical trials of type 2 diabetic patients to treat their complications [13]. This review seeks to briefly summarize the ample scientific literatures regarding curcumin as a potential treatment for diabetes and its associated complications. Particular attention will be given to the anti-inflammatory and antioxidant properties of curcumin.

## 2. Effect of Curcumin on Glycemia in Animal Model of Diabetes

Since Srinivasan discovered that curcumin has an effect on glycemia in one patient, a lot of papers have been published to discuss the ability of curcumin in controlling blood glucose in various rodent models (Table 1).

The most used animal in studying the effect of curcumin is the rat. Various diabetic rat models were employed to probe the effect of curcumin on glycemia. In alloxan-induced diabetes rats, streptozotocin- (STZ-) induced rats models, and STZ-nicotinamide-induced rats models [14], oral administration of various dosages of curcumin (80 mg/kg·body weight (BW) for 21 days [15] and 45 days [16]; 60 mg/kg·BW for 14 days [17]; 90 mg/kg·BW for 15 days [18]; 150 mg/kg·BW for 49 days [19]; 300 mg/kg·BW for 56 days [20]; 100 mg/kg·BW) for 4 weeks [21], 7 weeks [22], and 8 weeks [23] were able to prevent body weight loss, reduce the levels of glucose, hemoglobin (Hb), and glycosylated hemoglobin (HbA1C) in blood [15], and improve insulin sensitivity [16]. In addition, oral administration of turmeric aqueous extract (300 mg/kg·BW) [24] or curcumin (30 mg/kg·BW) for 56 days [25] resulted in a significant reduction in blood glucose in STZ-induced diabetes model in rats. In high fat diet (HFD) induced insulin resistance and type 2 diabetes models in rats, oral administration of curcumin (80 mg/kg·BW) for 15 and 60 days, respectively, showed an antihyperglycemic effect and improved insulin sensitivity [26]. Dietary curcumin (0.5% in diet) was also effective in ameliorating the increased levels of fasting blood glucose, urine sugar, and urine volume in STZ-induced diabetic rats [27].

Diabetic mice models were also employed to show the effect of curcumin on glycemia. In type 2 diabetic KK-A(y) mice, dietary turmeric extract (0.5% in diet, ethanol and/or hexane extraction) for 4 weeks significantly reduced the blood glucose levels [28]. In diet-induced obesity mice and *ob/ob* male mice, dietary curcumin (3%) for 6 weeks significantly improves glycemic status (blood glucose, glucose tolerance, and HbA1c) and insulin sensitivity [29]. In C57BL/KsJ *db/db* mice, dietary curcumin (0.2%) for 6 weeks was beneficial in improving glucose homeostasis and insulin resistance [30]. Curcumin (15 mg/kg·BW) for 30 days alone also suppressed elevated level of blood glucose in sodium arsenite treated rats [31]. In STZ-induced Swiss diabetic mice, intraperitoneal administration of curcumin (10 mM; 100 *μ*L/mouse) for 28 days significantly reversed hyperglycemia, glucose intolerance, and hypoinsulinemia [32]. In HFD induced obesity and insulin resistance mice, oral administration of curcumin (50 mg/kg·BW) for 15 days was effective in improving glucose intolerance [33].

The possible mechanisms of the effect of curcumin on glycemia in diabetes models may be explained as follows. First, curcumin could attenuate tumor necrosis factor-*α* (TNF-*α*) levels [32] and plasma free fatty acids (FFA) [26]. It also inhibits nuclear factor-kappa B (NF-*κ*B) activation [21] and protein carbonyl [34], lipid peroxidation [32], and lysosomal enzyme activities (N-acetyl-*β*-d-glucosaminidase, *β*-d-glucuronidase, *β*-d-galactosidase) [27]. In addition, curcumin can decrease the levels of thiobarbituric acid reactive substances (TBARS) and the activity of sorbitol dehydrogenase (SDH) [15, 24, 35]. Second, curcumin has the ability of induction of peroxisome proliferator-activated receptor-gamma (PPAR-*γ*) activation [28]. Curcumin also can elevate plasma insulin level and increase lipoprotein lipase (LPL) activity [30]. Third, curcumin is involved in activating of enzymes in liver, which are associated with glycolysis, gluconeogenic, and lipid metabolic process [30], and activating nuclear factor erythroid-2-related factor-2 (Nrf2) function as well [33].

Further, curcumin supplemented with vitamin C [20], yoghurt [36], and bone marrow transplantation [32] was effective in reducing the levels of blood glucose, Hb, and HbA1C in STZ diabetes models.

However, several researchers claimed that curcumin has no significant effect on blood glucose. Nishizono found that the intragastric administration of curcumin (200 mg/kg·BW) has no effect on serum concentration of glucose, insulin, and triacylglycerols in STZ- and HFD-induced diabetic Sprague Dawley rats for 14 days [37]. Majithiya also claimed that oral administration of curcumin from 4 weeks to 24 weeks (200 mg/kg·BW) has no significant effect on blood glucose and pressure in STZ diabetic rats [38]. The reason for yielding conflicting results from different groups may be due to different induction diabetes rodent models or different administration of curcumin.

## 3. Curcumin and Diabetes-Associated Liver Disorders

Diabetic patients often suffer from fatty liver disease and other liver disorders [39]. Babu and Srinivasan [40] found that STZ-induced diabetic rats fed dietary curcumin for 8 weeks excreted less albumin, urea, creatine, and inorganic phosphorus. Curcumin also reduced liver weight and lipid peroxidation products in the plasma and urine. In this study the beneficial effects of curcumin occurred independently of changes in glycemia or body weight. A further study by this group [41] suggested that hepatic cholesterol-7a-hydroxylase mediates the hypolipidemic action of curcumin in STZ diabetic rats. The effect of curcumin on lipidemia was also demonstrated by other groups [16, 20, 25, 36].

In sodium arsenite induced liver disorder rats, oral administration of curcumin can decrease total lipid, cholesterol, triglyceride (TG), and low density lipoprotein-cholesterol (LDL-c) [31].

Improved lipidemia by curcumin may be attributed to the induction of PPAR-*γ* activity [28, 42] that is linked to adipogenesis [43]. This improvement may also implicate the normalization of enzymatic activities [30] involved in lipid peroxidation [25] and glucose metabolism, including antioxidant enzymes (superoxide dismutase and catalase (SODC) and glutathione peroxidase (GPx)), hepatic glucose regulating enzymes (glucose-6-phosphatase(G6Pase), phosphoenolpyruvate carboxykinase (PEPCK)), hepatic lipid regulating enzymes (fatty acid synthase, 3-hydroxy-3-methylglutaryl coenzyme reductase, and acyl-CoA: cholesterol acyltransferase) [36], and malondialdehyde (MDA) [22, 38].

AMP-activated protein kinase (AMPK) is a strong energy regulator that controls whole-body glucose homeostasis in the liver and other key tissues in type 2 diabetes [44]. AMPK could stimulate glucose uptake and mediate suppression of hepatic gluconeogenesis. G6Pase and PEPCK are key enzymes involved in hepatic gluconeogenesis in the liver. Increased expression of G6Pase and PEPCK may have deleterious effects in diet-induced insulin resistance and type 2 diabetes [45]. Kim et al. [46] showed that curcumin inhibited PEPCK and G6Pase activities in H4IIE rat hepatoma and Hep3B human hepatoma cells. They further demonstrated that curcumin could increase phosphorylation of AMPK [47] and its downstream target acetyl-CoA carboxylase (ACC) [9] in H4IIE and Hep3B cells.

Hyperleptinemia associated with type 2 diabetes could cause hepatic fibrosis, which activates hepatic stellate cells (HSCs). As a sensor of cellular energy homeostasis, AMPK also stimulates fatty acid oxidation and regulates lipogenesis. Curcumin-mediated activation of AMPK could inactivate HSCs because of reduced stimulation by leptin [48], insulin, hyperglycemia [49], advanced glycation endproducts (AGEs) [50], and oxidized low-density lipoprotein (ox-LDL) [51]. The driving mechanisms behind hypolipidemia may be understood as follows. First, curcumin could disrupt insulin signaling and attenuate oxidative stress [52]. Second, curcumin could suppress membrane translocation and GLUT2-mediated gene expression. Third, curcumin was also able to increase expression of the AGE receptor [50], and reduce expression of lectin-like oxidized LDL receptor-1 (LOX-1) [51]. In addition, interruption of Wnt signaling [53] and stimulation of PPAR-*γ* activity [54] by curcumin can increase expression of genes involved in lipid accumulation.

Curcumin prevented liver fat accumulation in HFD rats. The anti-inflammatory and antilipolytic properties of curcumin may account for these results, as evident by reduced levels of TNF-*α* [55] and plasma FFA [26]. Further, curcumin normalized increased serum fetuin-A levels in HFD fed rats [56], while fetuin-A positively contributed to insulin resistance and fatty liver [57, 58].

In clinical trials, oral administration of low-dose curcumin (45 mg/day) for 2 months showed a trend of reduction in total cholesterol level and LDL cholesterol level in 63 acute coronary syndrome patients [59].

## 4. Curcumin and Adipose Tissue Dysfunction

Adipose tissue plays an important role in controlling wholebody glucose homeostasis [60]. Development of type 2 diabetes may involve deregulation of adiponectin secretion. Recent studies revealed that curcumin stimulated human adipocyte differentiation [7] and suppressed macrophage accumulation or activation in adipose tissue [61] by regulating adiponectin secretion [29, 62]. The mechanism may be due to suppression of NF-*κ*B activation [63], which reduces TNF-*α* and nitric oxide (NO) and inhibits the release of monocyte chemotactic protein-1 (MCP-1) from 3T3-L1 adipocytes [61]. Further studies also showed that suppression of 3T3-L1 adipocytes by curcumin was mediated through activation of Wnt/*β*-catenin signaling, which resulted in increased mRNA levels of c-Myc and cyclin D1 [64]. As is known to us, c-Myc and cyclin D1, well-known downstream target genes of *β*-catenin [65] [66], were shown to prevent adipogenesis [67, 68].

## 5. Curcumin and Diabetic Neuropathy

Diabetic neuropathy is neuropathic disorders that are associated with DM. These conditions are thought to result from diabetic microvascular injury, elevated AGEs, and activated protein kinase C (PKC) [69]. Curcumin has been actively involved in modulating the diabetic neuropathic disorders by the following lines of evidence. Curcumin effectively suppressed the development of diabetic cataracts in rat models of STZ-induced diabetes by reversing changes in lipid peroxidation, reduced glutathione, protein carbonyl content, and activities of antioxidant enzymes, which is beneficial to normalize expression of *α*A-crystallin and *α*B-crystallin [70, 71]. An increased expression of *α*A-crystallin and decreased expression of *α*B-crystallin were contributed to the reduction hydrophobicity and altered secondary and tertiary structures of acrystallin, which resulted in loss of neuroprotective function in diabetes [72, 73]. Suryanarayana et al. [74] also revealed that curcumin minimizes osmotic stress by regulating the polyol pathway. Further, hyperglycemia-induced aggregation and insolubilization of lens proteins were also prevented by curcumin.

Premanand et al. [75] showed that curcumin induces apoptosis of human retinal endothelial cells (HREC) by inhibiting vascular endothelial growth factor (VEGF) expression, intracellular reactive oxygen species (ROS) generation, and VEGF-mediated PKC-*β*2 translocation. Curcumin also exhibited an inhibitory effect on stromal-derived factor-1 (SDF-1) *α*-induced HREC migration by blocking upstream Ca(2+) influx and reducing downstream PI3K/Akt signals [76]. Curcumin may modulate antioxidant factors, including oxidatively modified DNA (8-OHdG), SODC, glutathione [77], and inflammatory parameters, including TNF-*α*, IL-1*β*, VEGF [78], and NF-*κ*B [79], and may also inhibit activation of nucleotide excision repair enzymes [80] in the retina of STZ-induced diabetic rats.

In addition, curcumin has been show to attenuate diabetes-induced cognitive deficits, as measured by the Morris water maze test [81], and cholinergic dysfunction involving acetylcholinesterase activity and cholinergic receptors [17, 82] through regulation of GLUT3, dopamine (D1, D2) receptors, CREB, phospholipase C [83], and insulin receptors [84]. These changes may be in part due to decreased glutamate-mediated excitotoxicity by curcumin, which alters the neurochemical parameters (NMDA and AMPA receptors) [85] in the cerebral cortices of diabetic rats. Curcumin reduced expression of single-minded 2 (Sim2) [86], which is involved in hyperglycemia-induced neuronal injury and impairment of learning and memory. Curcumin-mediated suppression of *β*-amyloid oligomers induces phosphorylation of tau and degradation of insulin receptor substrate via c-Jun N-terminal kinase (JNK) signaling in cultured hippocampal neurons, which is beneficial to improve cognitive deficits and insulin signaling in Alzheimer's disease [87]. Further, curcumin with/without gliclazide significantly attenuated diabetes-induced allodynia and hyperalgesia in STZ-induced diabetic mice [88] and rats [89, 90]. By virtue of its antioxidant and anti-inflammatory properties, the neuroprotective effects of curcumin are marked by alterations in MDA, total oxidant status, total antioxidant status, oxidative stress index, and NO [91] levels in the brain and sciatic tissues of diabetic rats [81, 92], which are mediated through regulation of TNF-*α* and TNF-*α* receptor [81, 89, 90].

## 6. Curcumin and Diabetic Nephropathy

Diabetic nephropathy is a clinical syndrome characterized by persistent albuminuria, progressive decline in the glomerular filtration rate, and elevated arterial blood pressure [93]. Currently, diabetic nephropathy is the leading cause of chronic kidney disease [94] and one of the most significant long-term complications in terms of morbidity and mortality for individual patients with diabetes. There are multiple mechanisms by which curcumin may ameliorate renal damage. Curcumin increases blood urea nitrogen [21, 95] and promotes clearance of creatine and urea [16, 96]. In addition, curcumin decreases levels of albuminuria [36, 76] and enzymuria, including levels of N-acetyl-D-glucosaminidase, lactate dehydrogenase (LDH), aspartate aminotransferase, alanine aminotransferase, and alkaline and acid phosphatases. Curcumin can also restore renal integrity by normalizing glutathione, SODC, glucose-6-phosphate dehydrogenase, LDH, aldose reductase, SDH, transaminases, ATPases, and membrane PUFA/SFA ratio [97]. A further study revealed that curcumin induces changes in posttranslational modification of histone H3 and altered expression of HSP-27 and p38 mitogen-activated protein kinase (MAPK) in diabetic kidneys [95]. These changes were mediated through inhibition of p300 and NF-*κ*B [98]. In addition, Ma et al. [99] reported that curcumin activated the p38-MAPK-HSP25 pathway in mouse podocytes but failed to attenuate albuminuria in STZ-induced diabetes in DBA2J mice. These mechanisms may be due to curcumin-mediated activation of AMP [100], which reduced expression of VEGF [101] and VEGF receptor, diminished the activities of PKC-*α* and PKC-*β*1 [23] and suppressed sterol regulatory element-binding protein (SREBP)-1c [100]. Clinical trials further confirmed the effect of curcumin on end-stage renal disease and showed that curcumin reduced transforming growth factor-*β* (TGF-*β*), IL-8, and urinary protein levels [102].

## 7. Curcumin and Diabetic Vascular Disease

Vascular disease is a common long-term complication of diabetes. Diabetic vascular disease causes damage to large and small blood vessels throughout the body. Curcumin has been reported to be active against diabetic vascular disease demonstrated by the following list of lines of evidence. First, curcumin modulated PKC-*α*, PKC-*β*2, and MAPK [103] and inhibited p300 [104] in experimental diabetic cardiomyopathy. Second, curcumin suppressed accelerated accumulation of AGE collagen and cross-linking of collagen in the tail tendon and skin of diabetic rats [105]. These effects were mediated by inhibition of VEGF [105], NF-*κ*B, and AP-1 [106]. Third, curcumin reduced endothelial nitric oxide synthase (eNOS) and inducible nitric oxide synthase (iNOS) levels, leading to less oxidative DNA and protein damage. This effect was also mediated by NF-*κ*B and AP-1 in diabetic rat hearts and microvascular endothelial cells stimulated with high glucose [107, 108]. Further studies by this group revealed that curcumin increased endothelin-1 levels. Fourth, curcumin improved diabetes-induced endothelial cell dysfunction through its antioxidant activity and PKC inhibition in STZ-induced diabetic rats [20] and mice [109]. Fifth, curcumin enhanced cutaneous wound healing in rats and guinea pigs [110]. A further study by this laboratory revealed that curcumin treatment mediated earlier reepithelialization, improved neovascularization, and increased migration of various cells, including dermal myofibroblasts, fibroblasts, and macrophages into the wound bed. These changes may have resulted from increased TGF-*β*1 levels. A recent study by Singh et al. [111] showed that insulin catalyzed curcumin-mediated wound healing by upregulating mitogenesis. The in vivo wound-healing capability of curcumin-loaded polycaprolactone nanofibers was demonstrated by an increased rate of wound closure in a STZ-induced mouse model of diabetes [112]. Sixth, curcumin prevented accumulation of AGEs [113] by trapping methylglyoxal [114] in human umbilical vein endothelial cells. Seventh, curcumin suppressed glycated-serum-albumin-(GSA-)induced IL-8 upregulation [115] via promoter activation and enhanced CXCL8 release in vascular smooth muscle cells. Eighth, curcumin attenuated diabetes-induced vascular dysfunction through inhibition of cyclooxygenase-2 (COX-2) activity, NF-*κ*B, and PKC and by improving the ratio of prostanoid products PGI(2)/TXA(2) in STZ rats [116]. Ninth, curcumin ameliorated exaggerated vascular contractility by reducing TNF-*α* and aortic ROS by inducing heme oxygenase-1 (HO-1) in hypertension-associated diabetic rat [117]. HO system plays an important role in triggering insulin release and modulating glucose metabolism [118, 119]. Curcumin treatment attenuated the phenylephrine-induced contraction and improved acetylcholine-induced relaxation in aortic ring in STZ diabetic rats [38]. Tenth, curcumin repaired and regenerated liver tissues by redeveloping liver microvasculars in diabetic rats [120]. Eleventh, a clinical trial showed that Meriva, a lecithinized formulation of curcumin, had beneficial effects on microcirculation and edema in diabetic microangiopathy [121] and retinopathy [122]. Twelfth, curcumin appeared to inhibit foam cell formation through the LOX-1 [123] pathway in human monocyte-derived macrophages in human diabetic atherosclerosis. Thirteenth, curcumin increased glucose utilization by preventing protein glycosylation and lipid peroxidation in erythrocytes exposed to high glucose [124]. This may be also due to the effect of curcumin on normalizing human erythrocyte membrane enzymes [30] and suppressing sorbitol accumulation through inhibition of aldose reductase activity [125]. Lastly, Pantazis et al. found that curcumin inhibited arsenic- (As(III)-) induced angiogenesis in human colon cancer cells and chicken chorioallantoic membrane model [126].

## 8. Curcumin and Other Diabetes-Associated Complications

The effects of curcumin on other diabetes-associated complications have been demonstrated by several studies. First, several groups demonstrated that curcumin was effective against diabetes-induced musculoskeletal diseases. Hie et al. [127] showed that curcumin suppressed diabetes-stimulated bone resorption by reducing tartrate-resistant acid phosphatase and cathepsin K, which was associated with inhibition of expression of c-fos and c-jun expression. The ability of curcumin to increase glucose uptake into skeletal muscle was mediated by improving the expressions of GLUT4 through the PLC-PI3K pathway [128] and insulin resistance in muscular tissue through the LKB1-AMPK pathway [19]. Curcumin effectively reduced the level of insulin receptor substrate-1 (IRS-1) phosphorylation on Ser307 and increased Akt phosphorylation [129] in skeletal muscle. In addition, curcumin and vitamin D3 reversed expression of *β*_2_-adrenoceptor, CREB, insulin receptor, Akt, and malate dehydrogenase activity in STZ-induced diabetic rat skeletal muscle almost to the levels observed in control samples [18].

Second, curcumin enhanced erectile function in diabetes-induced erectile dysfunction by increasing intracavernosal pressure (ICP), cGMP levels, HO-1, eNOS, neuronal NOS (nNOS), and Nrf2 with significant reductions in NF-*κ*B, p38, and iNOS [130]. Further, curcumin ameliorated STZ-induced testicular damage and apoptotic germ cell death by decreasing oxidative stress [131].

Finally, in diabetic gastroparesis rats, dietary curcumin for 6 weeks significantly improved gastric emptying rates as well as decreasing the levels of MDA and increasing SOD activity. The potential mechanism involved antioxidant action and enhancing expression of stem cell factor (SCF)/c-kit [132]. SCF/c-Kit signaling is important for recovering of the reducing interstitial cells of Cajal in diabetic gastroparesis in both humans and model animals [133, 134]. In B-lymphoma cells, curcumin-induced growth inhibition was mediated by reduced Akt activation and subsequent inhibition of spleen tyrosine kinase (Syk) [135].

## 9. Effect of Curcumin on Pancreatic *β*-Cell Dysfunction

The effect of curcumin on pancreatic cells has been extensively studied. First, curcumin increased islet viability and delayed islet ROS production, which is mediated through inhibiting poly ADP-ribose polymerase-1 activation (STZ-induced islet damage) [136] and normalizing cytokine (TNF*α*, IL-1*β*, and interferon-*γ*)-induced NF-*κ*B translocation by inhibiting phosphorylation of inhibitor of kappa B *α* (I*κ*B*α*) without affecting normal islet function in vitro, and by normalizing glucose clearance and pancreatic GLUT2 levels in STZ-treated mice [137]. Second, inclusion of curcumin in cryopreservation medium contributed to islet rescue by elevating HSP-70 and HO-1 [138]. Curcumin treatment increased the number of small pancreatic islets and decreased lymphocyte infiltration in pancreatic islets [139]. Inclusion of curcumin in bone marrow transplantation increased islet regeneration and insulin secretion [32]. Third, curcumin and its analogues played antioxidant defense by induction of the expression of HO-1, glutathione subunit, and NAD(P)H:quinone oxidoreductase 1 (antiapoptosis [140]) and increased basal insulin secretion in human islet [141], thus improving the outcome of islet transplantation. Fourth, curcumin increased the opening and activation of anion channels and depolarized the membrane potential, resulting in production of electronic activity and insulin release. Curcumin also decreased *β*-cell volume in rat pancreas [142]. Fifth, in a human pancreatic cell line, curcumin increased expression of the transcription factor 7-like 2 (*TCF7L2*) gene [143] in the Wnt signaling pathway, which is associated with type 2 diabetes [144]. Sixth, type 2 diabetes involved aberrant misfolding of human islet amyloid polypeptide (h-IAPP) and formation of pancreatic amyloid deposits [145]. Curcumin offered potential benefits by reducing h-IAPP fibril formation and aggregation [146], modulating IAPP self-assembly by unfolding *α*-helix [147], and inhibiting MCP-1-induced amylin mRNA expression [148]. All of the stimulatory actions of curcumin on pancreatic *β*-cells could contribute towards hypoglycemia in diabetes.

## 10. Curcumin and Its Anti-Inflammatory Actions

Inflammation is now recognized as one of the main contributors to diabetes and may be ameliorated by diminishing the underlying causes [149]. The beneficial effect of curcumin on diabetes may be due to its ability to spice up the immune system [150]. Margina et al. showed that curcumin restored transmembrane potential and stiffened membrane fluidity, limiting the release of proinflammatory factors, such as MCP-1 from endothelial and immune cells in human umbilical vein endothelial cells and Jurkat T lymphoblasts in the presence of high glucose or increased concentrations of AGEs [151]. These effects were more obvious during the late stages of diabetes.

Sharma et al. [152] showed that curcumin suppressed the activities of T- and B-lymphocytes and macrophages by inhibiting proliferation, antibody production (IgG1 and IgG2a), and lymphokine secretion (IL-4, IL-1, IL-6, and TNF-*α*) mainly by downregulating CD28 and CD80 and upregulating CTLA-4. In U937 monocytes, curcumin inhibited IL-6, IL-8, MCP-1, and TNF-*α* secretion in response to high glucose (35 mM). These effects were also reflected in STZ-induced diabetic rats, which exhibited significantly reduced blood levels of IL-6, MCP-1, TNF-*α*, glucose, HbA(1), and oxidative stress [22]. In addition, curcumin suppressed release of proinflammatory cytokines and histone acetylation in human monocytic (THP-1) cells, as demonstrated by increased activity of histone deacetylases (HDACs), reduced histone acetyltransferase (HAT) activity, reduced expression of p300 and acetylated CBP/p300, and altered NF-*κ*B binding [153]. Further, histone acetylation is an epigenetic modification. High glucose boosts production of cytokines via epigenetic changes, which are regulated through the opposing actions of HATs and HDACs. Dietary curcumin contributed to epigenetic modifications by regulating HATs and HDACs for diabetes prevention [154].

Curcumin treatment significantly inhibited degradation of I*κ*B*α* and NF-*κ*B activity, which is useful to reduce macrophage infiltration and prevent proinflammatory cytokines (TNF-*α* and IL-1*β*) from releasing and downregulate ICAM-1, MCP-1, and TGF-*β*1 protein expression in diabetic nephropathy [21].

Curcumin improved peripheral insulin resistance in insulin-resistant *ob/ob* mice with steatosis by reducing NF-*κ*B/RelA DNA-binding activity, decreasing mRNA level of TNF and IL-6, and enhancing IL-4 production in hepatic TNF/iNOS-producing dendritic cells and adipose tissue macrophages [155].

In high-fat diet-induced obese and leptin-deficient *ob/ob* mice, dietary curcumin ameliorated metabolic derangements by reversing many of inflammatory parameters, including reduced macrophage infiltration of white adipose tissue, increased adipose tissue adiponectin production, decreased hepatic NF-*κ*B activity, and hepatomegaly [29].

## 11. Curcumin and Its Antioxidant Actions

Increasing evidence demonstrates that increased levels of circulating ROS are involved in diabetes. Hyperglycemia causes autoxidation of glucose, glycation of proteins, and activation of polyol metabolism. These changes accelerate ROS generation and increase oxidative chemical modification of lipids, DNA, and proteins in various tissues [134]. Curcumin caused antioxidant effects through several mechanisms. First, curcumin dose-dependently abolished phorbol-12, myristate-13, acetate, and thapsigargin-induced ROS generation by inhibiting Ca2+ entry and PKC activity [156].

Second, curcumin blocked ROS formation, which led to cellular apoptosis by blocking subsequent apoptotic changes (DNA fragmentation, caspase-3 activation, cleavage of PARP, mitochondrial cytochrome c release, and JNK activation) in methylglyoxal-stimulated ESC-B5 cells, blastocysts, and human hepatoma G2 cells [157, 158].

Third, oral administration of photoirradiated curcumin resulted in near-normalization of antioxidant enzymatic activities and levels of lipid peroxidation markers, including circulatory lipid peroxidation, vitamin C, vitamin E, and SODC [141, 159].

Fourth, curcumin controlled oxidative stress by inhibiting increases in TBARS and protein carbonyls and reversing altered antioxidant enzyme activities in diabetic rats [34].

However, Majithiya and Balaraman [38] claimed that curcumin treatment had no significant effect on SODC and reduced glutathione levels. Curcumin treatment attenuated the phenylephrine-induced increase in contraction during the early stages of disease. However, this treatment had no significant effects during the medium and late stages. The reason why curcumin was unable to prevent oxidative stress is because of the excessive production of free radicals during the late stages.

## 12. Curcuminoids

Curcuminoids exhibit biological activities similar to those of curcumin [160] (Table 2). Curcuminoids derived from turmeric extract show significantly suppressed increasement in blood glucose levels by PPAR-*γ* activation and stimulated human adipocyte differentiation in type 2 diabetic KK-A(y) mice [28, 42]. Compared to curcumin, these synthesized curcuminoids have improved solubility and bioavailability [161–163]. The novel water-soluble curcumin derivative possesses antidiabetic actions, such as induction of HO, and improves the lipid profile with decreased lipid peroxides in the pancreas, liver, and aorta [164]. Curcuminoids improved diabetic complications in rat brains by accelerating antioxidant defense mechanisms and attenuating mitochondrial dysfunction [165].

Pugazhenthi et al. [166] showed that the further purification yields of curcumin, demethoxy curcumin (DMC), and bisdemethoxy curcumin (BDMC) induced expression of HO-1 through PI3K/Akt signaling in MIN6 cells. Real-time reverse transcription polymerase chain reaction also showed that DMC and BDMC elevated levels of glutamyl cysteine ligase (synthesis of glutathione) and NAD(P)H:quinone oxidoreductase (detoxifies quinines). Additional studies revealed that the induction was dependent on the presence of antioxidant response element (ARE) sites and the transcription factor that binds to ARE. Further, BDMC inactivated human pancreatic *α*-amylase [167], a therapeutic target for oral hypoglycemic agents in type 2 diabetes.

Osawa and Kato [168] showed that tetrahydrocurcumin (THC) scavenged ROS and increased glutathione concentrations in 25% galactose-fed SD rats with diabetic cataracts and in the cultured rat lens. Further studies revealed that THC normalized blood glucose by increasing plasma insulin, preventing lipid peroxidation (TBARS and hydroperoxides), and modulating levels of hepatic metabolic enzymes (hexokinase, glucose-6-phosphate dehydrogenase, fructose-1,6-bisphosphatase, and SDH) and antioxidant enzymes (SODC, GPx, glutathione-S-transferase, and reduced glutathione) in the liver, muscle, and brain of STZ-induced diabetic rats [14, 169]. THC also exhibited similar effects in STZ-nicotinamide-induced diabetic rats [170–173]. A further study by this laboratory showed that THC decreased the level of glycoprotein (hexose, hexosamine, fucose, and sialic acid) in diabetic rats [174]. In addition, THC normalized erythrocyte membrane-bounding enzymes [35], insulin receptor [175], renal abnormalities (urea, uric acid, and creatine) [16], and tail tendon collagen (accumulation and cross-linking of collagen) [176]. Further, combined treatment with THC and chlorogenic acid augmented enzymatic antioxidants and decreased lipid peroxidation [177] and blood glucose levels [178] in STZ-nicotinamide induced diabetic rats.

Reddy et al. [179, 180] discovered that bis-1,7-(2-hydroxyphenyl)-hepta-1,6-diene-3,5-dione, a BDMC analog, effectively decreased toxic effects and hyperlipidemia in STZ-nicotine induced diabetic rats. Bis-o-hydroxycinnamoylmethane, an analogue of the naturally occurring curcuminoid BDMC, exhibited antidiabetic properties by scavenging ROS production and protecting the pancreatic *β*-cell in hyperglycemic conditions [181].

Majithiya et al. [182] showed that the bis (curcumino) oxovanadium showed antidiabetic and hypolipidemic effects by decreasing blood glucose levels and serum lipids and restoring blood pressure and vascular reactivity to normal in STZ diabetic rats.

C66 and B06, two new synthetic analogues of curcumin, reduced production of TNF-*α* and NO, inhibited mRNA levels of IL-1*β*, TNF-*α*, IL-6, IL-12, COX-2, and iNOS, and inhibited activation of JNK/NF-*κ*B signaling in HG-stimulated primary peritoneal macrophages. C66 also improved histological abnormalities of kidney and heart but did not affect hyperglycemia in these diabetic rats [183, 184].

New formulation of curcumin has also been developed to improve its bioavailability. NCB-02, which is a standardized preparation of curcuminoids, had a favorable effect on endothelial dysfunction through anti-inflammatory and antioxidant mechanisms in a clinical trial [185].

## 13. Conclusion

**Figure 2:**
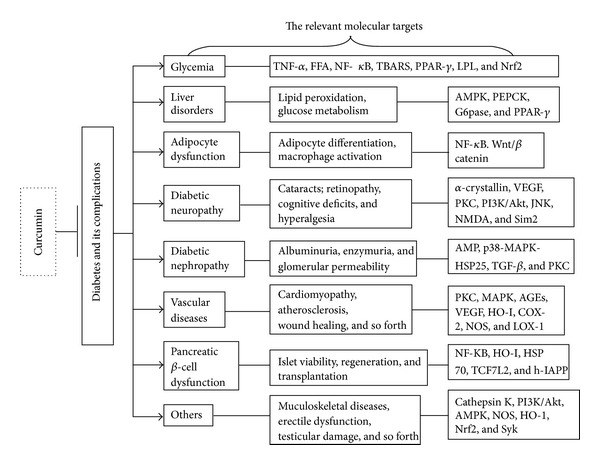
The relevant molecular targets of diabetes and its complications modulated by curcumin. Curcumin is actively involved in treating diabetes and diabetic disorders, which included liver disorders, adipocyte dysfunction, neuropathy, nephropathy, vascular diseases, pancreatic *β* cell dysfunction, and other complications. A lot of mediators and factors have been involved in the modulation process.

Recent research has provided the scientific basis for “traditional” curcumin and confirmed the important role of curcumin in the prevention and treatment of diabetes and its associated disorders. Curcumin could favorably affect most of the leading aspects of diabetes, including insulin resistance, hyperglycemia, hyperlipidemia, and islet apoptosis and necrosis (Figure 2). In addition, curcumin could prevent the deleterious complications of diabetes. Despite the potential tremendous benefits of this multifaceted nature product, results from clinical trials of curcumin are only available in using curcumin to treat diabetic nephropathy, microangiopathy and retinopathy so far. Studies are badly needed to be done in humans to confirm the potential of curcumin in limitation of diabetes and other associated disorders. Further, multiple approaches are also needed to overcome limited solubility and poor bioavailability of curcumin. These include synthesis of curcuminoids and development of novel formulations of curcumin, such as nanoparticles, liposomal encapsulation, emulsions, and sustained released tablets. Enhanced bioavailability and convinced clinical trial results of curcumin are likely to bring this promising natural product to the forefront of therapeutic agents for diabetes by generating a “super curcumin” in the near future.

## Figures and Tables

**Table 1:** Diabetic animal models employed in studying the effect of curcumin on glycemia.

Animal	Induction of diabetes (route and dose)	Curcumin (route and dose)	Course of treatment	Reference
Wistar rats	i.f. of STZ, 55 mg/kg·BW	Oral, 60 mg/kg·BW	14 days	[17, 18]
Wistar rats	i.p. of STZ, 55 mg/kg·BW; HFD	Oral, 150 mg/kg·BW	42 days	[19]
SD rats	HFD	Oral, 80 mg/kg·BW	15 and 60 days	[26]
SD rats	i.p. of STZ, 55 mg/kg·BW	Oral, 100 mg/kg·BW	28 days; 56 days	[21, 23]
Wistar rats	Injection of STZ, 45 mg/kg·BW	0.5% curcumin in diet	16 weeks	[27]
Wistar rats	i.p. of STZ, 55 mg/kg·BW	Oral, 300 mg/kg·BW	56 days	[20]
Wistar rats	i.p. of alloxan monohydrate (150 mg/kg·BW)	Oral, 80 mg/kg·BW	21 days	[15]
Swiss mice	i.p. of STZ (40 mg/kg·BW)	i.p., 10 mM	28 days	[32]
C57BL/6J mice	HFD	Oral, 50 mg/kg·BW	15 days	[33]
C57BL/6J mice: *ob/ob* mice	HFD	0.5% curcumin in diet	42 days	[29]
*db/db* mice	Not Applicable	0.02% curcumin in diet	42 days	[30]

i.f.: intrafemoral injection, i.p.: intraperitoneally injection.

**Table 2:** The applications of curcuminoids in treating diabetes and its associated disorders.

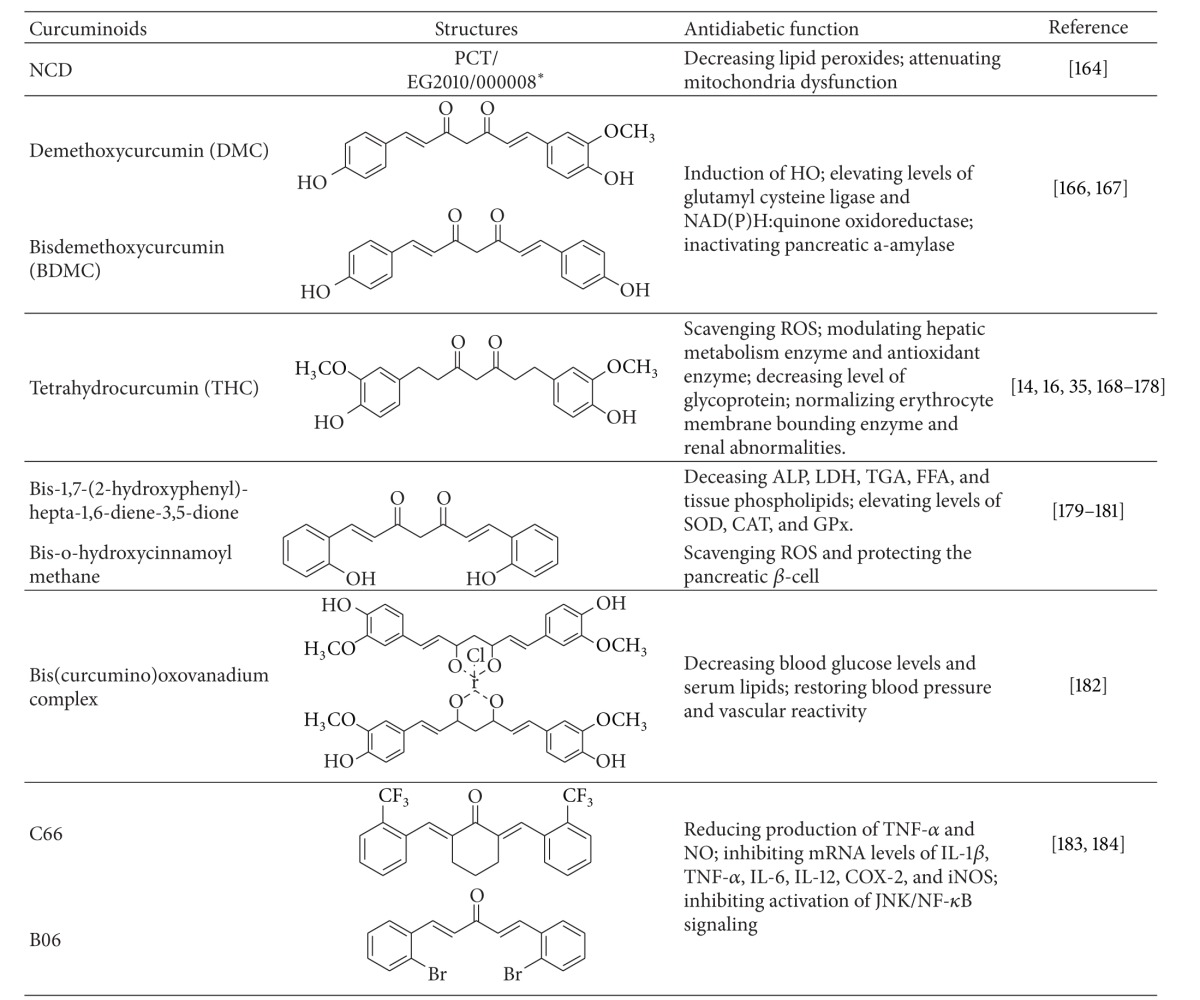

*Published patent pending, WO 2011/100984.

## References

[1] Shapiro K, Gong WC (2002). Natural products used for diabetes. *Journal of the American Pharmaceutical Association*.

[2] Gobert CP, Duncan AM (2009). Consumption, perceptions and knowledge of soy among adults with type 2 diabetes. *Journal of the American College of Nutrition*.

[3] Jiang CS, Liang LF, Guo YW (2012). Natural products possessing protein tyrosine phosphatase 1B (PTP1B) inhibitory activity found in the last decades. *Acta Pharmacologica Sinica*.

[4] Nolan CJ, Damm P, Prentki M (2011). Type 2 diabetes across generations: from pathophysiology to prevention and management. *The Lancet*.

[5] Aggarwal BB, Sundaram C, Malani N, Ichikawa H (2007). Curcumin: the Indian solid gold. *Advances in Experimental Medicine and Biology*.

[6] Kolev TM, Velcheva EA, Stamboliyska BA, Spiteller M (2005). DFT and experimental studies of the structure and vibrational spectra of curcumin. *International Journal of Quantum Chemistry*.

[7] Perez-Torres I, Ruiz-Ramirez A, Banos G, El-Hafidi M (2013). Hibiscus sabdariffa Linnaeus (Malvaceae), curcumin and resveratrol as alternative medicinal agents against metabolic syndrome. *Cardiovascular & Hematological Agents in Medicinal Chemistry*.

[8] Goel A, Kunnumakkara AB, Aggarwal BB (2008). Curcumin as “Curecumin”: from kitchen to clinic. *Biochemical Pharmacology*.

[9] Shehzad A, Ha T, Subhan F, Lee YS (2011). New mechanisms and the anti-inflammatory role of curcumin in obesity and obesity-related metabolic diseases. *European Journal of Nutrition*.

[10] Chuengsamarn S, Rattanamongkolgul S, Luechapudiporn R, Phisalaphong C, Jirawatnotai S (2012). Curcumin extract for prevention of type 2 diabetes. *Diabetes Care*.

[11] Aggarwal BB, Kumar A, Bharti AC (2003). Anticancer potential of curcumin: preclinical and clinical studies. *Anticancer Research*.

[12] Srinivasan M (1972). Effect of curcumin on blood sugar as seen in a diabetic subject. *Indian Journal of Medical Sciences*.

[13] Sahebkar A (2013). Why it is necessary to translate curcumin into clinical practice for the prevention and treatment of metabolic syndrome?. *BioFactors*.

[14] Pari L, Murugan P (2007). Tetrahydrocurcumin prevents brain lipid peroxidation in streptozotocin-induced diabetic rats. *Journal of Medicinal Food*.

[15] Arun N, Nalini N (2002). Efficacy of turmeric on blood sugar and polyol pathway in diabetic albino rats. *Plant Foods for Human Nutrition*.

[16] Murugan P, Pari L (2007). Influence of tetrahydrocurcumin on hepatic and renal functional markers and protein levels in experimental type 2 diabetic rats. *Basic and Clinical Pharmacology and Toxicology*.

[17] Peeyush KT, Gireesh G, Jobin M, Paulose CS (2009). Neuroprotective role of curcumin in the cerebellum of streptozotocin-induced diabetic rats. *Life Sciences*.

[18] Xavier S, Sadanandan J, George N, Paulose CS (2012). *β*
_2_-adrenoceptor and insulin receptor expression in the skeletal muscle of streptozotocin induced diabetic rats: antagonism by vitamin D_3_ and curcumin. *European Journal of Pharmacology*.

[19] Na L-X, Zhang Y-L, Li Y (2011). Curcumin improves insulin resistance in skeletal muscle of rats. *Nutrition, Metabolism and Cardiovascular Diseases*.

[20] Patumraj S, Wongeakin N, Sridulyakul P, Jariyapongskul A, Futrakul N, Bunnag S (2006). Combined effects of curcumin and vitamin C to protect endothelial dysfunction in the iris tissue of STZ-induced diabetic rats. *Clinical Hemorheology and Microcirculation*.

[21] Soetikno V, Sari FR, Veeraveedu PT (2011). Curcumin ameliorates macrophage infiltration by inhibiting NF-B activation and proinflammatory cytokines in streptozotocin induced-diabetic nephropathy. *Nutrition & Metabolism*.

[22] Jain SK, Rains J, Croad J, Larson B, Jones K (2009). Curcumin supplementation lowers TNF-*α*, IL-6, IL-8, and MCP-1 secretion in high glucose-treated cultured monocytes and blood levels of TNF-*α*, IL-6, MCP-1, glucose, and glycosylated hemoglobin in diabetic rats. *Antioxidants and Redox Signaling*.

[23] Soetikno V, Watanabe K, Sari FR (2011). Curcumin attenuates diabetic nephropathy by inhibiting PKC-*α* and PKC-*β*1 activity in streptozotocin-induced type I diabetic rats. *Molecular Nutrition and Food Research*.

[24] Ali Hussain HEM (2002). Hypoglycemic, hypolipidemic and antioxidant properties of combination of Curcumin from *Curcuma longa*, Linn, and partially purified product from Abroma augusta, Linn. in streptozotocin induced diabetes. *Indian Journal of Clinical Biochemistry*.

[25] Mahesh T, Sri Balasubashini MM, Menon VP (2004). Photo-irradiated curcumin supplementation in streptozotocin-induced diabetic rats: effect on lipid peroxidation. *Therapie*.

[26] El-Moselhy MA, Taye A, Sharkawi SS, El-Sisi SFI, Ahmed AF (2011). The antihyperglycemic effect of curcumin in high fat diet fed rats. Role of TNF-*α* and free fatty acids. *Food and Chemical Toxicology*.

[27] Chougala MB, Bhaskar JJ, Rajan MGR, Salimath PV (2012). Effect of curcumin and quercetin on lysosomal enzyme activities in streptozotocin-induced diabetic rats. *Clinical Nutrition*.

[28] Nishiyama T, Mae T, Kishida H (2005). Curcuminoids and sesquiterpenoids in turmeric (*Curcuma longa* L.) Suppress an increase in blood glucose level in type 2 diabetic KK-A*γ* mice. *Journal of Agricultural and Food Chemistry*.

[29] Weisberg SP, Leibel R, Tortoriello DV (2008). Dietary curcumin significantly improves obesity-associated inflammation and diabetes in mouse models of diabesity. *Endocrinology*.

[30] Seo K-I, Choi M-S, Jung UJ (2008). Effect of curcumin supplementation on blood glucose, plasma insulin, and glucose homeostasis related enzyme activities in diabetic db/db mice. *Molecular Nutrition and Food Research*.

[31] Yousef MI, El-Demerdash FM, Radwan FME (2008). Sodium arsenite induced biochemical perturbations in rats: ameliorating effect of curcumin. *Food and Chemical Toxicology*.

[32] El-Azab MF, Attia FM, El-Mowafy AM (2011). Novel role of curcumin combined with bone marrow transplantation in reversing experimental diabetes: effects on pancreatic islet regeneration, oxidative stress, and inflammatory cytokines. *European Journal of Pharmacology*.

[33] He HJ, Wang GY, Gao Y, Ling WH, Yu ZW, Jin TR (2012). Curcumin attenuates Nrf2 signaling defect, oxidative stress in muscle and glucose intolerance in high fat diet-fed mice. *World Journal of Diabetes*.

[34] Suryanarayana P, Satyanarayana A, Balakrishna N, Kumar PU, Bhanuprakash Reddy G (2007). Effect of turmeric and curcumin on oxidative stress and antioxidant enzymes in streptozotocin-induced diabetic rat. *Medical Science Monitor*.

[35] Murugan P, Pari L (2007). Influence of tetrahydrocurcumin on erythrocyte membrane bound enzymes and antioxidant status in experimental type 2 diabetic rats. *Journal of Ethnopharmacology*.

[36] Gutierres VO, Pinheiro CM, Assis RP, Vendramini RC, Pepato MT, Brunetti IL (2012). Curcumin-supplemented yoghurt improves physiological and biochemical markers of experimental diabetes. *The British Journal of Nutrition*.

[37] Nishizono S, Hayami T, Ikeda I, Imaizumi K (2000). Protection against the diabetogenic effect of feeding tert-butylhydroquinone to rats prior to the administration of streptozotocin. *Bioscience, Biotechnology and Biochemistry*.

[38] Majithiya JB, Balaraman R (2005). Time-dependent changes in antioxidant enzymes and vascular reactivity of aorta in streptozotocin-induced diabetic rats treated with curcumin. *Journal of Cardiovascular Pharmacology*.

[39] Prentki M, Madiraju SRM (2008). Glycerolipid metabolism and signaling in health and disease. *Endocrine Reviews*.

[40] Babu PS, Srinivasan K (1995). Influence of dietary curcumin and cholesterol on the progression of experimentally induced diabetes in albino rat. *Molecular and Cellular Biochemistry*.

[41] Babu PS, Srinivasan K (1997). Hypolipidemic action of curcumin, the active principle of turmeric (*Curcuma longa*) in streptozotocin induced diabetic rats. *Molecular and Cellular Biochemistry*.

[42] Kuroda M, Mimaki Y, Nishiyama T (2005). Hypoglycemic effects of turmeric (*Curcuma longa* L. rhizomes) on genetically diabetic KK-Ay mice. *Biological and Pharmaceutical Bulletin*.

[43] Deng T, Sieglaff DH, Zhang A (2011). A peroxisome proliferator-activated receptor *γ* (PPAR*γ*)/ PPAR*γ* coactivator 1*β* autoregulatory loop in adipocyte mitochondrial function. *The Journal of Biological Chemistry*.

[44] Schultze SM, Hemmings BA, Niessen M, Tschopp O (2012). PI3K/AKT, MAPK and AMPK signalling: protein kinases in glucose homeostasis. *Expert Reviews in Molecular Medicine*.

[45] Franckhauser S, Muñoz S, Elias I, Ferre T, Bosch F (2006). Adipose overexpression of phosphoenolpyruvate carboxykinase leads to high susceptibility to diet-induced insulin resistance and obesity. *Diabetes*.

[46] Kim T, Davis J, Zhang AJ, He X, Mathews ST (2009). Curcumin activates AMPK and suppresses gluconeogenic gene expression in hepatoma cells. *Biochemical and Biophysical Research Communications*.

[47] Fujiwara H, Hosokawa M, Zhou X (2008). Curcumin inhibits glucose production in isolated mice hepatocytes. *Diabetes Research and Clinical Practice*.

[48] Tang Y, Chen A (2010). Curcumin protects hepatic stellate cells against leptin-induced activation in vitro by accumulating intracellular lipids. *Endocrinology*.

[49] Lin J, Chen A (2011). Curcumin diminishes the impacts of hyperglycemia on the activation of hepatic stellate cells by suppressing membrane translocation and gene expression of glucose transporter-2. *Molecular and Cellular Endocrinology*.

[50] Lin J, Tang Y, Kang Q, Feng Y, Chen A (2012). Curcumin inhibits gene expression of receptor for advanced glycation end-products (RAGE) in hepatic stellate cells in vitro by elevating PPARgamma activity and attenuating oxidative stress. *British Journal of Pharmacology*.

[51] Kang Q, Chen A (2009). Curcumin eliminates oxidized LDL roles in activating hepatic stellate cells by suppressing gene expression of lectin-like oxidized LDL receptor-1. *Laboratory Investigation*.

[52] Lin J, Zheng S, Chen A (2009). Curcumin attenuates the effects of insulin on stimulating hepatic stellate cell activation by interrupting insulin signaling and attenuating oxidative stress. *Laboratory Investigation*.

[53] Gustafson B, Smith U (2006). Cytokines promote Wnt signaling and inflammation and impair the normal differentiation and lipid accumulation in 3T3-L1 preadipocytes. *The Journal of Biological Chemistry*.

[54] Hegarty BD, Furler SM, Ye J, Cooney GJ, Kraegen EW (2003). The role of intramuscular lipid in insulin resistance. *Acta Physiologica Scandinavica*.

[55] Xie XY, Kong PR, Wu JF, Li Y, Li YX (2012). Curcumin attenuates lipolysis stimulated by tumor necrosis factor-alpha or isoproterenol in 3T-L1 adipocytes. *Phytomedicine*.

[56] Oner-Iyidogan Y, Kocak H, Seyidhanoglu M (2013). Curcumin prevents liver fat accumulation and serum fetuin-A increase in rats fed a high-fat diet. *Journal of Physiology and Biochemistry*.

[57] Haukeland JW, Dahl TB, Yndestad A (2012). Fetuin A in nonalcoholic fatty liver disease: in vivo and in vitro studies. *European Journal of Endocrinology*.

[58] Stefan N, Hennige AM, Staiger H (2006). *α*2-Heremans-Schmid glycoprotein/fetuin-A is associated with insulin resistance and fat accumulation in the liver in humans. *Diabetes Care*.

[59] Alwi I, Santoso T, Suyono S (2008). The effect of curcumin on lipid level in patients with acute coronary syndrome. *Acta medica Indonesiana*.

[60] Guilherme A, Virbasius JV, Puri V, Czech MP (2008). Adipocyte dysfunctions linking obesity to insulin resistance and type 2 diabetes. *Nature Reviews Molecular Cell Biology*.

[61] Woo H-M, Kang J-H, Kawada T, Yoo H, Sung M-K, Yu R (2007). Active spice-derived components can inhibit inflammatory responses of adipose tissue in obesity by suppressing inflammatory actions of macrophages and release of monocyte chemoattractant protein-1 from adipocytes. *Life Sciences*.

[62] Ohara K, Uchida A, Nagasaka R, Ushio H, Ohshima T (2009). The effects of hydroxycinnamic acid derivatives on adiponectin secretion. *Phytomedicine*.

[63] Gonzales AM, Orlando RA (2008). Curcumin and resveratrol inhibit nuclear factor-kappaB-mediated cytokine expression in adipocytes. *Nutrition & Metabolism*.

[64] Ahn J, Lee H, Kim S, Ha T (2010). Curcumin-induced suppression of adipogenic differentiation is accompanied by activation of Wnt/*β*-catenin signaling. *American Journal of Physiology*.

[65] He T-C, Sparks AB, Rago C (1998). Identification of c-MYC as a target of the APC pathway. *Science*.

[66] Tetsu O, McCormick F (1999). *β*-catenin regulates expression of cyclin D1 in colon carcinoma cells. *Nature*.

[67] Ninomiya-Tsuji J, Torti FM, Ringold GM (1993). Tumor necrosis factor-induced c-myc expression in the absence of mitogenesis is associated with inhibition of adipocyte differentiation. *Proceedings of the National Academy of Sciences of the United States of America*.

[68] Fu M, Rao M, Bouras T (2005). Cyclin D1 inhibits peroxisome proliferator-activated receptor *γ*-mediated adipogenesis through histone deacetylase recruitment. *The Journal of Biological Chemistry*.

[69] Joshi RP, Negi G, Kumar A (2013). SNEDDS curcumin formulation leads to enhanced protection from pain and functional deficits associated with diabetic neuropathy: an insight into its mechanism for neuroprotection. *Nanomedicine: Nanotechnology, Biology and Medicine *.

[70] Kumar PA, Suryanarayana P, Reddy PY, Reddy GB (2005). Modulation of *α*-crystallin chaperone activity in diabetic rat lens by curcumin. *Molecular Vision*.

[71] Kumar PA, Haseeb A, Suryanarayana P, Ehtesham NZ, Reddy GB (2005). Elevated expression of *α*A- and *α*B-crystallins in streptozotocin-induced diabetic rat. *Archives of Biochemistry and Biophysics*.

[72] Kase S, Ishida S, Rao NA (2011). Increased expression of *α*A-crystallin in human diabetic eye. *International Journal of Molecular Medicine*.

[73] Losiewicz MK, Fort PE (2011). Diabetes impairs the neuroprotective properties of retinal alpha-crystallins. *Investigative Ophthalmology & Visual Science*.

[74] Suryanarayana P, Saraswat M, Mrudula T, Krishna TP, Krishnaswamy K, Reddy GB (2005). Curcumin and turmeric delay streptozotocin-induced diabetic cataract in rats. *Investigative Ophthalmology and Visual Science*.

[75] Premanand C, Rema M, Sameer MZ, Sujatha M, Balasubramanyam M (2006). Effect of curcumin on proliferation of human retinal endothelial cells under in vitro conditions. *Investigative Ophthalmology and Visual Science*.

[76] Sameermahmood Z, Balasubramanyam M, Saravanan T, Rema M (2008). Curcumin modulates SDF-1*α*/CXCR4-induced migration of human retinal endothelial cells (HRECs). *Investigative Ophthalmology and Visual Science*.

[77] Gupta SK, Kumar B, Nag TC (2011). Curcumin prevents experimental diabetic retinopathy in rats through its hypoglycemic, antioxidant, and anti-inflammatory mechanisms. *Journal of Ocular Pharmacology and Therapeutics*.

[78] Mrudula T, Suryanarayana P, Srinivas PNBS, Reddy GB (2007). Effect of curcumin on hyperglycemia-induced vascular endothelial growth factor expression in streptozotocin-induced diabetic rat retina. *Biochemical and Biophysical Research Communications*.

[79] Kowluru RA, Kanwar M (2007). Effects of curcumin on retinal oxidative stress and inflammation in diabetes. *Nutrition & Metabolism*.

[80] Wang C, George B, Chen S, Feng B, Li X, Chakrabarti S (2012). Genotoxic stress and activation of novel DNA repair enzymes in human endothelial cells and in the retinas and kidneys of streptozotocin diabetic rats. *Diabetes/Metabolism Research and Reviews*.

[81] Kuhad A, Chopra K (2007). Curcumin attenuates diabetic encephalopathy in rats: behavioral and biochemical evidences. *European Journal of Pharmacology*.

[82] Peeyush Kumar T, Antony S, Soman S, Kuruvilla KP, George N, Paulose CS (2011). Role of curcumin in the prevention of cholinergic mediated cortical dysfunctions in streptozotocin-induced diabetic rats. *Molecular and Cellular Endocrinology*.

[83] Kumar TP, Antony S, Gireesh G, George N, Paulose CS (2010). Curcumin modulates dopaminergic receptor, CREB and phospholipase C gene expression in the cerebral cortex and cerebellum of streptozotocin induced diabetic rats. *Journal of Biomedical Science*.

[84] Kumar PT, George N, Antony S, Skaria Paulose C (2013). Curcumin restores diabetes induced neurochemical changes in the brain stem of Wistar rats. *European Journal of Pharmacology*.

[85] Jayanarayanan S, Smijin S, Peeyush KT, Anju TR, Paulose CS (2013). NMDA and AMPA receptor mediated excitotoxicity in cerebral cortex of streptozotocin induced diabetic rat: ameliorating effects of curcumin. *Chemico-Biological Interactions*.

[86] Wang X, Song Y, Chen L (2013). Contribution of single-minded 2 to hyperglycaemia-induced neurotoxicity. *Neurotoxicology*.

[87] Ma Q-L, Yang F, Rosario ER (2009). *β*-Amyloid oligomers induce phosphorylation of tau and inactivation of insulin receptor substrate via c-Jun N-terminal kinase signaling: suppression by omega-3 fatty acids and curcumin. *Journal of Neuroscience*.

[88] Sharma S, Kulkarni SK, Agrewala JN, Chopra K (2006). Curcumin attenuates thermal hyperalgesia in a diabetic mouse model of neuropathic pain. *European Journal of Pharmacology*.

[89] Attia HN, Al-Rasheed NM, Al-Rasheed NM, Maklad YA, Ahmed AAE, Kenawy SAB (2012). Protective effects of combined therapy of gliclazide with curcumin in experimental diabetic neuropathy in rats. *Behavioural Pharmacology*.

[90] Li Y, Zhang Y, Liu DB, Liu HY, Hou WG, Dong YS (2013). Curcumin attenuates diabetic neuropathic pain by downregulating TNF-*α* in a rat model. *International Journal of Medical Sciences*.

[91] Sharma S, Chopra K, Kulkarni SK (2007). Effect of insulin and its combination with resveratrol or curcumin in attenuation of diabetic neuropathic pain: participation of nitric oxide and TNF-alpha. *Phytotherapy Research*.

[92] Acar A, Akil E, Alp H (2012). Oxidative damage is ameliorated by curcumin treatment in brain and sciatic nerve of diabetic rats. *The International Journal of Neuroscience*.

[93] Maric-Bilkan C (2013). Obesity and diabetic kidney disease. *The Medical Clinics of North America*.

[94] Reutens AT (2013). Epidemiology of diabetic kidney disease. *The Medical Clinics of North America*.

[95] Tikoo K, Meena RL, Kabra DG, Gaikwad AB (2008). Change in post-translational modifications of histone H3, heat-shock protein-27 and MAP kinase p38 expression by curcumin in streptozotocin-induced type I diabetic nephropathy. *British Journal of Pharmacology*.

[96] Sharma S, Kulkarni SK, Chopra K (2006). Curcumin, the active principle of turmeric (*Curcuma longa*), ameliorates diabetic nephropathy in rats. *Clinical and Experimental Pharmacology and Physiology*.

[97] Babu PS, Srinivasan K (1998). Amelioration of renal lesions associated with diabetes by dietary curcumin in streptozotocin diabetic rats. *Molecular and Cellular Biochemistry*.

[98] Chiu J, Khan ZA, Farhangkhoee H, Chakrabarti S (2009). Curcumin prevents diabetes-associated abnormalities in the kidneys by inhibiting p300 and nuclear factor-*κ*B. *Nutrition*.

[99] Ma J, Phillips L, Wang Y (2010). Curcumin activates the p38MPAK-HSP25 pathway in vitro but fails to attenuate diabetic nephropathy in DBA2J mice despite urinary clearance documented by HPLC. *BMC Complementary and Alternative Medicine*.

[100] Soetikno V, Sari FR, Sukumaran V (2013). Curcumin decreases renal triglyceride accumulation through AMPK-SREBP signaling pathway in streptozotocin-induced type 1 diabetic rats. *The Journal of Nutritional Biochemistry*.

[101] Sawatpanich T, Petpiboolthai H, Punyarachun B, Anupunpisit V (2010). Effect of curcumin on vascular endothelial growth factor expression in diabetic mice kidney induced by streptozotocin. *Journal of the Medical Association of Thailand = Chotmaihet Thangphaet*.

[102] Khajehdehi P, Pakfetrat M, Javidnia K (2011). Oral supplementation of turmeric attenuates proteinuria, transforming growth factor-*β* and interleukin-8 levels in patients with overt type 2 diabetic nephropathy: a randomized, double-blind and placebo-controlled study. *Scandinavian Journal of Urology and Nephrology*.

[103] Soetikno V, Sari FR, Sukumaran V (2012). Curcumin prevents diabetic cardiomyopathy in streptozotocin-induced diabetic rats: possible involvement of PKC-MAPK signaling pathway. *European Journal of Pharmaceutical Sciences*.

[104] Feng B, Chen S, Chiu J, George B, Chakrabarti S (2008). Regulation of cardiomyocyte hypertrophy in diabetes at the transcriptional level. *American Journal of Physiology*.

[105] Sajithlal GB, Chithra P, Chandrakasan G (1998). Effect of curcumin on the advanced glycation and cross-linking of collagen in diabetic rats. *Biochemical Pharmacology*.

[106] Okamoto T, Yamagishi S-I, Inagaki Y (2002). Angiogenesis induced by advanced glycation end products and its prevention by cerivastatin. *The FASEB Journal*.

[107] Farhangkhoee H, Khan ZA, Chen S, Chakrabarti S (2006). Differential effects of curcumin on vasoactive factors in the diabetic rat heart. *Nutrition & Metabolism*.

[108] Srivastava G, Mehta JL (2009). Currying the heart: curcumin and cardioprotection. *Journal of Cardiovascular Pharmacology and Therapeutics*.

[109] Rungseesantivanon S, Thenchaisri N, Ruangvejvorachai P, Patumraj S (2010). Curcumin supplementation could improve diabetes-induced endothelial dysfunction associated with decreased vascular superoxide production and PKC inhibition. *BMC Complementary and Alternative Medicine*.

[110] Sidhu GS, Mani H, Gaddipati JP (1999). Curcumin enhances wound healing in streptozotocin induced diabetic rats and genetically diabetic mice. *Wound Repair and Regeneration*.

[111] Singh N, Ranjan V, Zaidi D (2012). Insulin catalyzes the curcumin-induced wound healing: an in vitro model for gingival repair. *Indian Journal of Pharmacology*.

[112] Merrell JG, McLaughlin SW, Tie L, Laurencin CT, Chen AF, Nair LS (2009). Curcumin-loaded poly(*ε*-caprolactone) nanofibres: diabetic wound dressing with anti-oxidant and anti-inflammatory properties. *Clinical and Experimental Pharmacology and Physiology*.

[113] Elosta A, Ghous T, Ahmed N (2012). Natural products as Anti-glycation agents: possible therapeutic potential for diabetic complications. *Current Diabetes Reviews*.

[114] Hu TY, Liu CL, Chyau CC, Hu ML (2012). Trapping of methylglyoxal by curcumin in cell-free systems and in human umbilical vein endothelial cells. *Journal of Agricultural and Food Chemistry*.

[115] Choi K-H, Park J-W, Kim H-Y (2010). Cellular factors involved in CXCL8 expression induced by glycated serum albumin in vascular smooth muscle cells. *Atherosclerosis*.

[116] Rungseesantivanon S, Thengchaisri N, Ruangvejvorachai P, Patumraj S (2010). Curcumin improves prostanoid ratio in diabetic mesenteric arteries associated with cyclooxygenase-2 and NF-*κ*B suppression. *Diabetes, Metabolic Syndrome and Obesity: Targets and Therapy*.

[117] Hassan N, El-Bassossy HM, Zakaria MN (2013). Heme oxygenase-1 induction protects against hypertension associated with diabetes: effect on exaggerated vascular contractility. *Naunyn-Schmiedeberg's Archives of Pharmacology*.

[118] Ndisang JF, Jadhav A (2009). Heme oxygenase system enhances insulin sensitivity and glucose metabolism in streptozotocin-induced diabetes. *American Journal of Physiology*.

[119] Ndisang JF, Jadhav A (2010). The heme oxygenase system attenuates pancreatic lesions and improves insulin sensitivity and glucose metabolism in deoxycorticosterone acetate hypertension. *American Journal of Physiology*.

[120] Khimmaktong W, Petpiboolthai H, Panyarachun B, Anupunpisit V (2012). Study of curcumin on microvasculature characteristic in diabetic rat’s liver as revealed by vascular corrosion cast/scanning electron microscope (SEM) technique. *Journal of the Medical Association of Thailand = Chotmaihet Thangphaet*.

[121] Appendino G, Belcaro G, Cornelli U (2011). Potential role of curcumin phytosome (Meriva) in controlling the evolution of diabetic microangiopathy. A pilot study. *Panminerva Medica*.

[122] Steigerwalt R, Nebbioso M, Appendino G (2012). Meriva, a lecithinized curcumin delivery system, in diabetic microangiopathy and retinopathy. *Panminerva Medica*.

[123] Li L, Sawamura T, Renier G (2004). Glucose enhances human macrophage LOX-1 expression: role for LOX-1 in glucose-induced macrophage foam cell formation. *Circulation Research*.

[124] Jain SK, Rains J, Jones K (2006). Effect of curcumin on protein glycosylation, lipid peroxidation, and oxygen radical generation in human red blood cells exposed to high glucose levels. *Free Radical Biology and Medicine*.

[125] Muthenna P, Suryanarayana P, Gunda SK, Petrash JM, Reddy GB (2009). Inhibition of aldose reductase by dietary antioxidant curcumin: mechanism of inhibition, specificity and significance. *FEBS Letters*.

[126] Pantazis P, Varman A, Simpson-Durand C (2010). Curcumin and turmeric attenuate arsenic-induced angiogenesis in ovo. *Alternative Therapies in Health and Medicine*.

[127] Hie M, Yamazaki M, Tsukamoto I (2009). Curcumin suppresses increased bone resorption by inhibiting osteoclastogenesis in rats with streptozotocin-induced diabetes. *European Journal of Pharmacology*.

[128] Cheng T-C, Lin C-S, Hsu C-C, Chen L-J, Cheng K-C, Cheng J-T (2009). Activation of muscarinic M-1 cholinoceptors by curcumin to increase glucose uptake into skeletal muscle isolated from Wistar rats. *Neuroscience Letters*.

[129] Deng Y-T, Chang T-W, Lee M-S, Lin J-K (2012). Suppression of free fatty acid-induced insulin resistance by phytopolyphenols in C2C12 mouse skeletal muscle cells. *Journal of Agricultural and Food Chemistry*.

[130] Abdel Aziz MT, Motawi T, Rezq A (2012). Effects of a water-soluble curcumin protein conjugate vs. pure curcumin in a diabetic model of erectile dysfunction. *Journal of Sexual Medicine*.

[131] Kanter M, Aktas C, Erboga M (2012). Curcumin attenuates testicular damage, apoptotic germ cell death, and oxidative stress in streptozotocin-induced diabetic rats. *Molecular Nutrition & Food Research*.

[132] Jin QH, Shen HX, Wang H, Shou QY, Liu Q (2013). Curcumin improves expression of SCF/c-kit through attenuating oxidative stress and NF-*κ*B activation in gastric tissues of diabetic gastroparesis rats. *Diabetology & Metabolic Syndrome*.

[133] Iwasaki H, Kajimura M, Osawa S (2006). A deficiency of gastric interstitial cells of Cajal accompanied by decreased expression of neuronal nitric oxide synthase and substance P in patients with type 2 diabetes mellitus. *Journal of Gastroenterology*.

[134] Giacco F, Brownlee M (2010). Oxidative stress and diabetic complications. *Circulation Research*.

[135] Gururajan M, Dasu T, Shahidain S (2007). Spleen tyrosine kinase (Syk), a novel target of curcumin, is required for B lymphoma growth. *Journal of Immunology*.

[136] Meghana K, Sanjeev G, Ramesh B (2007). Curcumin prevents streptozotocin-induced islet damage by scavenging free radicals: a prophylactic and protective role. *European Journal of Pharmacology*.

[137] Kanitkar M, Gokhale K, Galande S, Bhonde RR (2008). Novel role of curcumin in the prevention of cytokine-induced islet death in vitro and diabetogenesis in vivo. *British Journal of Pharmacology*.

[138] Kanitkar M, Bhonde RR (2008). Curcumin treatment enhances islet recovery by induction of heat shock response proteins, Hsp70 and heme oxygenase-1, during cryopreservation. *Life Sciences*.

[139] Chanpoo M, Petchpiboonthai H, Panyarachun B, Anupunpisit V (2010). Effect of curcumin in the amelioration of pancreatic islets in streptozotocin-induced diabetic mice. *Journal of the Medical Association of Thailand = Chotmaihet Thangphaet*.

[140] Zafar KS, Inayat-Hussain SH, Siegel D, Bao A, Shieh B, Ross D (2006). Overexpression of NQO1 protects human SK-N-MC neuroblastoma cells against dopamine-induced cell death. *Toxicology Letters*.

[141] Balamurugan AN, Akhov L, Selvaraj G, Pugazhenthi S (2009). Induction of antioxidant enzymes by curcumin and its analogues in human islets: implications in transplantation. *Pancreas*.

[142] Best L, Elliott AC, Brown PD (2007). Curcumin induces electrical activity in rat pancreatic *β*-cells by activating the volume-regulated anion channel. *Biochemical Pharmacology*.

[143] Khalooghi K, Hashemi S, Mehraban N (2009). In vitro modulation of TCF7L2 gene expression in human pancreatic cells. *Molecular Biology Reports*.

[144] Yan Y, Klein R, Heiss G (2010). The transcription factor 7-like 2 (TCF7L2) polymorphism may be associated with focal arteriolar narrowing in Caucasians with hypertension or without diabetes: the ARIC Study. *BMC Endocrine Disorders*.

[145] Ran C, Zhao W, Moir RD, Moore A (2011). Non-conjugated small molecule FRET for differentiating monomers from higher molecular weight amyloid beta species. *PLoS ONE*.

[146] Daval M, Bedrood S, Gurlo T (2010). The effect of curcumin on human islet amyloid polypeptide misfolding and toxicity. *Amyloid*.

[147] Sparks S, Liu G, Robbins KJ, Lazo ND (2012). Curcumin modulates the self-assembly of the islet amyloid polypeptide by disassembling alpha-helix. *Biochemical and Biophysical Research Communications*.

[148] Cai K, Qi D, Hou X (2011). MCP-1 upregulates amylin expression in murine pancreatic *β* cells through ERK/JNK-AP1 and NF-*κ*B related signaling pathways independent of CCR2. *PLoS ONE*.

[149] Xie W, Du L (2011). Diabetes is an inflammatory disease: evidence from traditional Chinese medicines. *Diabetes, Obesity and Metabolism*.

[150] Jagetia GC, Aggarwal BB (2007). “Spicing up” of the immune system by curcumin. *Journal of Clinical Immunology*.

[151] Margina D, Gradinaru D, Manda G, Neagoe I, Ilie M (2013). Membranar effects exerted in vitro by polyphenols—quercetin, epigallocatechin gallate and curcumin—on HUVEC and Jurkat cells, relevant for diabetes mellitus. *Food and Chemical Toxicology*.

[152] Sharma S, Chopra K, Kulkarni SK, Agrewala JN (2007). Resveratrol and curcumin suppress immune response through CD28/CTLA-4 and CD80 co-stimulatory pathway. *Clinical and Experimental Immunology*.

[153] Yun J-M, Jialal I, Devaraj S (2011). Epigenetic regulation of high glucose-induced proinflammatory cytokine production in monocytes by curcumin. *Journal of Nutritional Biochemistry*.

[154] Pham TX, Lee J (2012). Dietary regulation of histone acetylases and deacetylases for the prevention of metabolic diseases. *Nutrients*.

[155] Yekollu SK, Thomas R, O’Sullivan B (2011). Targeting curcusomes to inflammatory dendritic cells inhibits NF-*κ*B and improves insulin resistance in obese mice. *Diabetes*.

[156] Balasubramanyam M, Koteswari AA, Kumar RS, Monickaraj SF, Maheswari JU, Mohan V (2003). Curcumin-induced inhibition of cellular reactive oxygen species generation: novel therapeutic implications. *Journal of Biosciences*.

[157] Hsuuw Y-D, Chang C-K, Chan W-H, Yu J-S (2005). Curcumin prevents methylglyoxal-induced oxidative stress and apoptosis in mouse embryonic stem cells and blastocysts. *Journal of Cellular Physiology*.

[158] Chan W-H, Wu H-J, Hsuuw Y-D (2005). Curcumin inhibits ROS formation and apoptosis in methylglyoxal-treated human hepatoma G2 cells. *Annals of the New York Academy of Sciences*.

[159] Mahesh T, Balasubashini MS, Menon VP (2005). Effect of photo-irradiated curcumin treatment against oxidative stress in streptozotocin-induced diabetic rats. *Journal of Medicinal Food*.

[160] Fan X, Zhang C, Liu DB, Yan J, Liang HP (2013). The clinical applications of curcumin: current state and the future. *Current Pharmaceutical Design*.

[161] Yang CS, Sang S, Lambert JD, Lee M-J (2008). Bioavailability issues in studying the health effects of plant polyphenolic compounds. *Molecular Nutrition and Food Research*.

[162] Anand P, Thomas SG, Kunnumakkara AB (2008). Biological activities of curcumin and its analogues (Congeners) made by man and Mother Nature. *Biochemical Pharmacology*.

[163] Gupta SC, Patchva S, Aggarwal BB (2013). Therapeutic roles of curcumin: lessons learned from clinical trials. *The AAPS Journal*.

[164] Abdel Aziz MT, El-Asmar MF, El-Ibrashy IN (2012). Effect of novel water soluble curcumin derivative on experimental type-1 diabetes mellitus (short term study). *Diabetology & Metabolic Syndrome*.

[165] Rastogi M, Ojha R, Rajamanickam GV, Agrawal A, Aggarwal A, Dubey GP (2008). Curcuminoids modulates oxidative damage and mitochondrial dysfunction in diabetic rat brain. *Free Radical Research*.

[166] Pugazhenthi S, Akhov L, Selvaraj G, Wang M, Alam J (2007). Regulation of heme oxygenase-1 expression by demethoxy curcuminoids through Nrf2 by a PI3-kinase/Akt-mediated pathway in mouse *β*-cells. *American Journal of Physiology*.

[167] Ponnusamy S, Zinjarde S, Bhargava S, Rajamohanan PR, Ravikumar A (2012). Discovering Bisdemethoxycurcumin from *Curcuma longa* rhizome as a potent small molecule inhibitor of human pancreatic alpha-amylase, a target for type-2 diabetes. *Food Chemistry*.

[168] Osawa T, Kato Y (2005). Protective role of antioxidative food factors in oxidative stress caused by hyperglycemia. *Annals of the New York Academy of Sciences*.

[169] Pari L, Murugan P (2005). Effect of tetrahydrocurcumin on blood glucose, plasma insulin and hepatic key enzymes in streptozotocin induced diabetic rats. *Journal of Basic and Clinical Physiology and Pharmacology*.

[170] Murugan P, Pari L (2006). Antioxidant effect of tetrahydrocurcumin in streptozotocin-nicotinamide induced diabetic rats. *Life Sciences*.

[171] Murugan P, Pari L (2006). Effect of tetrahydrocurcumin on plasma antioxidants in streptozotocin-nicotinamide experimental diabetes. *Journal of Basic and Clinical Physiology and Pharmacology*.

[172] Murugan P, Pari L (2006). Effect of tetrahydrocurcumin on lipid peroxidation and lipids in streptozotocin-nicotinamide-induced diabetic rats. *Basic and Clinical Pharmacology and Toxicology*.

[173] Pari L, Murugan P (2007). Antihyperlipidemic effect of curcumin and tetrahydrocurcumin in experimental type 2 diabetic rats. *Renal Failure*.

[174] Pari L, Murugan P (2007). Changes in glycoprotein components in streptozotocin—nicotinamide induced type 2 diabetes: influence of tetrahydrocurcumin from *Curcuma longa*. *Plant Foods for Human Nutrition*.

[175] Murugan P, Pari L, Rao CA (2008). Effect of tetrahydrocurcumin on insulin receptor status in type 2 diabetic rats: studies on insulin binding to erythrocytes. *Journal of Biosciences*.

[176] Pari L, Murugan P (2007). Influence of tetrahydrocurcumin on tail tendon collagen contents and its properties in rats with streptozotocin-nicotinamide-induced type 2 diabetes. *Fundamental and Clinical Pharmacology*.

[177] Pari L, Karthikesan K, Menon VP (2010). Comparative and combined effect of chlorogenic acid and tetrahydrocurcumin on antioxidant disparities in chemical induced experimental diabetes. *Molecular and Cellular Biochemistry*.

[178] Karthikesan K, Pari L, Menon VP (2010). Combined treatment of tetrahydrocurcumin and chlorogenic acid exerts potential antihyperglycemic effect on streptozotocin-nicotinamide-induced diabetic rats. *General Physiology and Biophysics*.

[179] Reddy BV, Sundari JS, Balamurugan E, Menon VP (2009). Prevention of nicotine and streptozotocin treatment induced circulatory oxidative stress by bis-1,7-(2-hydroxyphenyl)-hepta-1,6-diene-3,5-dione in diabetic rats. *Molecular and Cellular Biochemistry*.

[180] Reddy BV, Sivagama Sundari J, Balamurugan E, Menon VP (2010). Antihyperlipidemic effect of bis-1,7-(2-hydroxyphenyl)-hepta-1,6-diene-3,5-dione, a curcumin analog, on nicotine and streptozotocin treated rats. *Molecular and Cellular Biochemistry*.

[181] Srinivasan A, Menon VP, Periaswamy V, Rajasekaran KN (2003). Protection of pancreatic *β*-cell by the potential antioxidant bis-o-hydroxycinnamoyl methane, analogue of natural curcuminoid in experimental diabetes. *Journal of Pharmacy & Pharmaceutical Sciences*.

[182] Majithiya JB, Balaraman R, Giridhar R, Yadav MR (2005). Effect of bis[curcumino]oxovanadium complex on non-diabetic and streptozotocin-induced diabetic rats. *Journal of Trace Elements in Medicine and Biology*.

[183] Pan Y, Wang Y, Cai L (2012). Inhibition of high glucose-induced inflammatory response and macrophage infiltration by a novel curcumin derivative prevents renal injury in diabetic rats. *British Journal of Pharmacology*.

[184] Pan Y, Zhu G, Wang Y (2013). Attenuation of high-glucose-induced inflammatory response by a novel curcumin derivative B06 contributes to its protection from diabetic pathogenic changes in rat kidney and heart. *The Journal of Nutritional Biochemistry*.

[185] Usharani P, Mateen AA, Naidu MUR, Raju YSN, Chandra N (2008). Effect of NCB-02, atorvastatin and placebo on endothelial function, oxidative stress and inflammatory markers in patients with type 2 diabetes mellitus: a randomized, parallel-group, placebo-controlled, 8-week study. *Drugs in R&D*.

